# Improvement in rheumatic fever and rheumatic heart disease management and prevention using a health centre-based continuous quality improvement approach

**DOI:** 10.1186/1472-6963-13-525

**Published:** 2013-12-18

**Authors:** Anna P Ralph, Marea Fittock, Rosalie Schultz, Dale Thompson, Michelle Dowden, Tom Clemens, Matthew G Parnaby, Michele Clark, Malcolm I McDonald, Keith N Edwards, Jonathan R Carapetis, Ross S Bailie

**Affiliations:** 1 Menzies School of Health Research, Darwin, Northern Territory (NT), Australia; 2 Division of Medicine, Royal Darwin Hospital, Darwin, NT, Australia; 3 Nyangirru Piliyi-ngara Kurantta, Anyinginyi Health Aboriginal Corporation, Tennant Creek, NT, Australia; 4 Ngalkanbuy Health Service, Galiwinku, NT, Australia; 5 Northern Territory Department of Health and Community Services, Townsville, Australia; 6 Queensland Health, Queensland Government, Townsville, Queensland, Australia; 7 School of Medicine and Dentistry, Cairns Campus, James Cook University, Townsville, QLD, Australia; 8 Telethon Institute for Child Health Research, Centre for Child Health Research, University of Western Australia, Perth, Western Australia, Australia

**Keywords:** Continuous quality improvement, Rheumatic fever, Rheumatic heart disease, Secondary prophylaxis

## Abstract

**Background:**

Rheumatic heart disease (RHD) remains a major health concern for Aboriginal Australians. A key component of RHD control is prevention of recurrent acute rheumatic fever (ARF) using long-term secondary prophylaxis with intramuscular benzathine penicillin (BPG). This is the most important and cost-effective step in RHD control. However, there are significant challenges to effective implementation of secondary prophylaxis programs. This project aimed to increase understanding and improve quality of RHD care through development and implementation of a continuous quality improvement (CQI) strategy.

**Methods:**

We used a CQI strategy to promote implementation of national best-practice ARF/RHD management guidelines at primary health care level in Indigenous communities of the Northern Territory (NT), Australia, 2008–2010. Participatory action research methods were employed to identify system barriers to delivery of high quality care. This entailed facilitated discussion with primary care staff aided by a system assessment tool (SAT). Participants were encouraged to develop and implement strategies to overcome identified barriers, including better record-keeping, triage systems and strategies for patient follow-up. To assess performance, clinical records were audited at baseline, then annually for two years. Key performance indicators included proportion of people receiving adequate secondary prophylaxis (≥80% of scheduled 4-weekly penicillin injections) and quality of documentation.

**Results:**

Six health centres participated, servicing approximately 154 people with ARF/RHD. Improvements occurred in indicators of service delivery including proportion of people receiving ≥40% of their scheduled BPG (increasing from 81/116 [70%] at baseline to 84/103 [82%] in year three, p = 0.04), proportion of people reviewed by a doctor within the past two years (112/154 [73%] and 134/156 [86%], p = 0.003), and proportion of people who received influenza vaccination (57/154 [37%] to 86/156 [55%], p = 0.001). However, the proportion receiving ≥80% of scheduled BPG did not change. Documentation in medical files improved: ARF episode documentation increased from 31/55 (56%) to 50/62 (81%) (p = 0.004), and RHD risk category documentation from 87/154 (56%) to 103/145 (76%) (p < 0.001). Large differences in performance were noted between health centres, reflected to some extent in SAT scores.

**Conclusions:**

A CQI process using a systems approach and participatory action research methodology can significantly improve delivery of ARF/RHD care.

## Background

Acute rheumatic fever (ARF) and rheumatic heart disease (RHD) remain important causes of illness and premature death among young people in low-resource settings [1], and among minority Indigenous populations in Australia, New Zealand and elsewhere [2-6]. Aboriginal Australians are almost 20 times more likely to die from ARF and RHD than other Australians, with 40% of deaths occurring in people aged ≤35 years [7]. RHD causes most of the excess morbidity and mortality attributable to ARF [8]. While valve damage can occur after a single episode of ARF, most RHD results from cumulative valvular insults brought by recurrent episodes of ARF [9].

Primordial prevention of ARF targets social determinants, particularly household crowding [3]. Primary prevention is directed towards group A streptococcal infection, either through antimicrobial treatment and/or vaccination. As yet, no effective vaccine is available [10]. Secondary prevention entails long-term, sometimes life-long, 3–4 weekly intramuscular injections of benzathine penicillin G (BPG) to prevent recurrent ARF in people with a history of ARF or known RHD. Tertiary management addresses the clinical consequences of established RHD [11-14].

Sustained reductions in ARF incidence could be achieved by improvements in living conditions [9,15]. In the meantime, significant reductions in disease burden can be best achieved through successful implementation of secondary ARF prevention programs [16,17]. Primary prevention using antibiotics for treatment of streptococcal pharyngitis has proved to be of limited sustainability in remote Aboriginal communities in the northern 'Top End’ of Australia’s Northern Territory (NT), largely because of the resource commitment required. Symptomatic pharyngitis is also rare despite the high rates of ARF [18-21]. Secondary prevention of ARF with long-term 3–4 weekly BPG injections (Table 1) has been proven to work and is cost-effective [17,22]. However, this strategy requires a well-functioning, properly-resourced and highly-motivated primary health service. The logistics of distance are often formidable. The focus must be on children, adolescents and young adults; and the injections are painful. Factors which intuitively would be thought to be associated with adherence likelihood, such as pain of needles and knowledge of ARF/RHD, are in fact not necessarily the main determinants of adherence in our setting [23]. Long-term daily oral penicillin is not acceptably effective [24], or feasible in remote settings. Rates of penicillin allergy are fortunately low, with oral erythromycin used in these few instances [13]. School-based delivery of injections has proven to be very efficacious in New Zealand [25], but is a limited option in our setting due to low school attendance, recognised challenges in the implementation of school-based health programs in remote communities, and the requirement for ongoing prophylaxis for many people after school-leaving age.

**Table 1:** **Rheumatic heart disease severity grading and indication for secondary prophylaxis **[13]

**Risk classification**	**Clinical description**	**Duration of secondary prophylaxis**
Low risk	History of acute rheumatic fever with no evidence of rheumatic heart disease OR trivial to mild valvular disease.	Min 10 yrs after episode of ARF or age 21, whichever is longer
Medium risk	Moderate valve lesion in the absence of symptoms and with normal left ventricular function OR mechanical prosthetic valves.	Until age 35
High risk	Severe valvular disease OR moderate/severe valvular lesion with symptoms OR tissue prosthetic valves and valve repairs.	Until age 40, or longer if ongoing exposure to GAS remains high and risk also considered very high

Effective control of RHD first requires active engagement of primary health care staff backed up by a well-designed and maintained ARF/RHD register. There is international consensus that register-based programs are essential for effective delivery of secondary prophylaxis at the community level and for coordinating follow-up [12,16,23]. In a setting of high medical and nursing staff turnover, cultural disparities, vast distances and harsh climatic conditions, sustainability of any chronic disease program is the great challenge.

A critical measure of an effective secondary prevention program is the rate of ARF recurrence. When a register-based secondary prevention program was introduced in the NT Top End in 1997 [26], around 45% of ARF cases were recurrences [27]. By 2006, this proportion had fallen to 30% (Top End RHD Control Program – unpublished data). In New Zealand, where ARF/RHD registers have been operating for many years, documented recurrence rates are less than 10% [28]. In a 2005 study of ARF prevention in a large NT remote Aboriginal community, less than half the people on the local register received 80% or more of their scheduled BPG doses in the preceding 12 months, and more than half had missed follow-up echocardiograms or specialist appointments [29]. There is still much to be done before service delivery can be regarded as acceptable across the region.

There has been strong uptake of systematic approaches to continuous quality improvement (CQI) in the NT [30] and in Indigenous primary health care services elsewhere across Australia [31]. This has mainly been driven by the Audit and Best Practice for Chronic Disease (ABCD) project and the subsequent establishment of the National Centre for Quality Improvement in Indigenous Primary Health Care (One21seventy) [32]. CQI methods facilitate proper collection and subsequent application of primary health performance data. The primary mechanism is direct and active engagement of primary health staff. Such CQI strategies are well-suited to Australian Indigenous primary health settings [30] and have been shown to enhance health centre systems, delivery of quality clinical care and various intermediate outcomes [33]. An improved model of health service delivery has already been shown to be an effective strategy in reducing ARF rates; four decades ago, ARF rates fell in parts of Baltimore, USA, between 1960–64 and 1968–70, in areas in which a 'comprehensive-care program’ was implemented, compared with the rest of Baltimore [34].

Evidence-based guidelines for ARF/RHD diagnosis and management [13,35] outline the basis for ARF/RHD management nationally and internationally. These address all aspects of ARF/RHD management from primordial prevention to tertiary management of valvular lesions; their recommendations on secondary prophylaxis inform this study. Little is known about the extent of the gap between best practice and actual practice across this scope of ARF/RHD care. The aims of this project were to enhance understanding of the gap between best practice and actual practice in prevention and management of RHD, and to improve ARF/RHD management through development and implementation of a structured systems approach in collaboration with remote NT Indigenous health services. This involved integrating the principles of the 2006 ARF/RHD National Guidelines [35] into the ABCD program [36,37].

## Methods

### Study setting and processes

The primary health clinics were in regional and remote Aboriginal communities in the Australia’s Northern Territory (Top End and Central Australia). A project management committee was established comprising the lead project investigators, health service managers, clinicians, staff of the NT RHD Control Program, and staff of RHD Australia [http://www.rhdaustralia.org.au/]. Participant consent was not required for this quality improvement audit. Ethics approval was given by the Health Research Ethics Committees of the Northern Territory Department of Health, Top End and Central Australia.

### Development and implementation of CQI intervention

**Figure 1:**
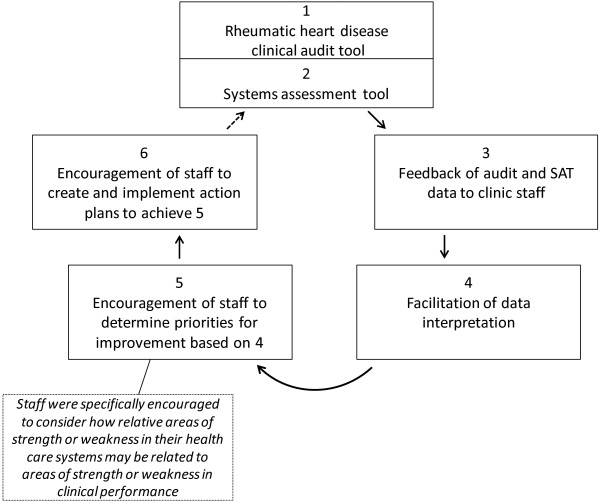
Continuous quality improvement cycle.

The first step was to specifically modify the pre-existing ABCD processes and tools [32,37] for the ARF/RHD project. The CQI intervention was delivered through an action research design, whereby health centre staff, RHD Control Program staff and other key stakeholders were involved in the development of the tools. Experience gained by participants and results of regular data feedback were then used to continually revise and improve the tools. This action research process aimed to maximise engagement and support of stakeholders at all levels, from management to clinic primary care staff and support personnel. The cyclical CQI process is summarised in Figure 1. The CQI process has two essential components: the RHD clinical audit tool for data collection from clinical records and the ABCD Systems Assessment Tool for staff to score their own performance.

### RHD clinical audit tool

The project officer and local health centre staff used the clinical audit tool (Additional file 1) and audit protocol to assess the relevant clinical records of people with known RHD or at least one episode of suspected or confirmed ARF, who were known to have resided in the community for at least six of the 12 preceding months. RHD risk category (low, medium or high risk based on extent of rheumatic valvular damage, see Table 1) was assigned by the project officer on the basis of available clinical information, unless already documented in the clinical record. The key indicators of clinical performance were determined by the evidence-based RHD guidelines [35] and expert opinion of medical specialists experienced in RHD and Aboriginal health. Audits were conducted at baseline and annually for the next two years. Delivery of ≥80% of scheduled BPG doses in the preceding 12 months is one recommended minimum benchmark, [13] although it is well known that recurrent ARF can occur after just one missed BPG injection.

### ABCD systems assessment tool

The ABCD Systems Assessment Tool (SAT) (Table 2) was used to assess the clinic systems required to support best practice in prevention and management of ARF/ RHD. It comprised an interactive process whereby a facilitator engaged health centre staff (Aboriginal Health Workers, nurses, doctors, administrative and other non-clinical staff) in discussion and reflection on the strengths and weaknesses of their health centre. They were encouraged to consider the systems currently in place to support best practice. A scoring tool (available via http://www.one21seventy.org.au), was then completed as part of the process. Each health centre had annual systems assessment with the SAT over the 3-year project. The same facilitator conducted each SAT in order to better standardise scoring between health centres and over the project’s course. The process of determining SAT scores was a key component of the action research methodology and was not primarily intended to provide an objective measure of health centre performance, but rather to stimulate reflection by the health centre team about potential for system improvement. The determinants of relatively good or relatively poor performance were identified through a thematic analysis of data drawn from these discussions and observations by the project officer.

**Table 2:** Components of the acute rheumatic fever and rheumatic heart disease services systems assessment tool

**Domain**	**Components**
Delivery system design	- team structure and function
	- clinical leadership
	- appointments and scheduling
	- care planning
	- systematic follow-up
	- continuity of care
	- client access and cultural competence
	- infrastructure, supplies and equipment
Information systems and decision support	- maintenance and use of electronic client lists
	- evidence based guidelines
	- specialist – generalist collaborations
Self-management support	- assessment and documentation
	- education and support
Links with the community, other health services and other services and resources	- cooperation on governance and operations
	- linking health service clients to other resources
	- working out in the community
	- cooperation on regional health planning and resource development
Organisational influence and integration	- organisational commitment
	- quality improvement strategies
	- integration of systems in health centre

*(formatted here as a list due to space constraints. See*http://www.one21seventy.org.au*for formatted version created for data collection).*

### Statistical methods

Data entry, analysis and reporting for the CQI process were supported by the One21seventy web-based information system [32]. We estimated that 6 NT health centres, covering approximately 150 people with ARF/RHD, would provide an appropriately diverse range to ensure transferability and external validity of the findings. Analyses were performed using Stata 10.0 (Stata Corporation, College Station, TX, USA). Chi-squared tests for trend were used to test differences in proportions.

## Results

### Characteristics of participating health centres

From January 2008 to December 2010, six remote Aboriginal health centres were engaged in the study. Table 3 shows their diversity in terms of population size, geography, accessibility, staffing, record keeping and governance arrangements.

**Table 3:** Characteristics of participating health care centres

**Health centre**	**Approximate population**	**Approximate number of people with ARF/RHD**	**Location of health centre**	**Climate (tropical, sub-tropical, desert)**	**Management: (Aboriginal medical service [AMS], Government)**	**Record keeping (Electronic, paper, both)**	**Full-time doctor (Yes, No)**
A	1160	24	Remote community, 6 hour drive on mostly sealed road	Desert	AMS	Both	No (year 1)
							Yes (years 2–3)
B	600	18	Remote community, 2 hour drive on mostly unsealed road	Desert	AMS	Both	Yes
C	1500	30	Remote island community, 2–3 hours flying time to major service centre; weekly barge service	Tropical	Government	Paper	No
D	Main community – 115	21	Remote community, 2.5 hour drive on sealed road to major service centre	Sub-tropical	Government	Both (transition from paper to electronic during study)	No
	Including outstations serviced by clinic - 600						
E	9022	42	Regional service centre	Tropical	AMS	Electronic	Yes
F	990	14	Remote community, 20 minute drive to small town	Tropical	Government	Paper	No

The number of clinical records audited in each of the three years was 154 in 2008, 145 in 2009 and 156 in 2010. Factors accounting for year-to-year variation in numbers were: 1. People moving into and out of communities participating in the project; 2. New ARF/RHD diagnoses - 6 occurred in 4 communities; 3. Deaths in people with ARF/RHD - 5 in 2 communities; 4. Inability to locate the clinical file at the time of audit.

In the baseline audit, 136/154 (88%) of people were aged ≥15 years, 12% 5–15 years and none <5 years; 95/154 (62%) were female, and 99% had Indigenous status recorded as Aboriginal (152 people) or Torres Strait Islander (1 person). Over the three years <1% of clinical records did not have Indigenous status recorded.

### Variation between clinics

**Figure 2:**
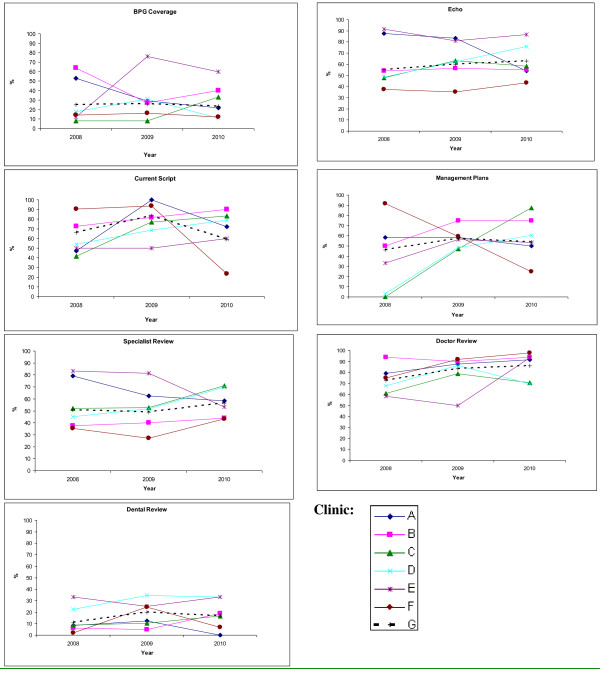
**Trends in key indicators.** BPG Coverage = % patients receiving ≥80% of scheduled injections; Current script =% patients with a current BPG prescription on file; Management Plans refers to % patients with a current management plan in the clinical record; Doctor review (and Specialist review) refer to % patients with a record of having their health and RHD care reviewed by a doctor (specialist) within a specified period in relation to RHD risk status; Echo =% patients with a record of having an echocardiogram within a specified period in relation to RHD risk status; Dental review = % patients with a record of having a dental review within two years of the audit date. Solid lines each show data for a specific health centre (as identified by the letters in the legend). The dashed line shows the aggregate data for the six health centres.

There was wide variation in achievement of key performance indicators between clinics. Proportions of people who received ≥80% of scheduled BPG injections at baseline ranged from <10% in one clinic to >70% at another with an average across clinics of 25% [29/116] (see Figure 2). The proportion of people for whom risk classification was documented at different health centres ranged from 25 to 100% in year one of the study.

### Risk classification

Overall, approximately 40% of people were in the medium to high-risk RHD category; 70/154 (45%) in 2008, 52/145 (36%) in 2009, 61/156 (39%) in 2010. For 84-85% of people it was possible to determine the category from documentation in clinical record or through the auditor applying an algorithm based on data in the clinical record. The proportion of people with a diagnosis of RHD documented in their clinical record that were identified as requiring secondary prophylaxis decreased over the study from 75% in 2008 to 67% in 2009 and 66% in 2010. This trend followed clinical review by a specialist paediatrician, internal medicine physician or cardiologist. Among the subgroup of people in the low-risk category, the proportion dropped from 83% in 2008 to 63% in 2010.

Overall, there was frequent contact with the community health centre; >80% of all people with RHD, including those not requiring regular BPG, attended the clinic in the 3 months prior to each audit. People in the high and medium-risk categories were more likely to have attended within the preceding month than those in low or undetermined-risk categories (Table 4). The most common reason cited for attending was ARF/RHD care. The health practitioner who conducted the first assessment was usually a nurse (41%-52% of people), followed by an Aboriginal Health Worker (30-42%), then a General Practitioner (12-14%).

**Table 4:** **Documentation of rheumatic heart disease information in health centre clinical records of people with ARF/RHD**^1^

	**Risk classification**	**2008**	**2009**	**2010**	**p value**
Diagnosis recorded on Client’s clinical record summary sheet	Recurrent or suspected recurrent ARF episode	56% (31/55)	73% (44/60)	81% (50/62)	**0.004**
	Rheumatic heart disease	84% (115/137)	86% (115/133)	90% (135/150)	0.12
Documentation of risk classification in full clinical record	All	56% (87/154)	71% (103/145)	76% (118/156)	**<0.001**
	High/Med	36% (50/138)	43% (45/104)	46% (56/122)	0.11
Documentation of risk classification in the clinical record summary sheet page?	All	29% (44/154)	34% (50/145)	56% (88/156)	**<0.001**
	High/Med	35% (24/69)	42% (22/52)	64% (39/61)	**0.001**
ARF/RHD management plan in notes	All	46% (71/154)	57% (83/145)	53% (83/156)	0.22
	High/Med	51% (35/69)	77% (40/52)	62% (38/61)	0.15
	Low/Undetermined	42% (36/85)	46% (43/93)	47% (45/95)	0.50
Current prescription on file	All	66% (77/116)	82% (81/99)	58% (60/103)	0.24
	High/Med	72% (41/57)	82% (36/44)	64% (30/47)	0.41
	Low/Undetermined	61% (36/59)	82% (45/55)	54% (30/56)	0.43
Smoking status recorded	All	23% (36/154)	40% (58/145)	38% (60/156)	**0.005**
Attendance within the previous month	All	68% (105/154)	63% (92/145)	65% (101/156)	0.53
	High/Med	71% (49/69)	62% (32/52)	80% (49/61)	0.27
	Low/Undetermined	66% (56/85)	65% (60/93)	55% (52/95)	0.56
Attendance within the previous three months	All	84% (129/154)	90% (130/145)	86% (134/156)	0.59
	High/Med	86% (59/69)	90% (47/52)	93% (57/61)	0.14
	Low/Undetermined	82% (70/85)	89% (83/93)	81% (77/95)	0.77

^1^Except for the two indicators “Documentation of risk classification in clinical record” and “Documentation of risk classification in the clinical record summary sheet” all risk classifications are based on documented risk classification where available or, if there was no clear documented risk classification, assessment by auditor applying an algorithm to clinical data available in the record.

Bold text is used to highlight p-values of <0.05.

### Impact of the CQI process on documentation and health care attendance

When compared with baseline, significantly more people had documentation of recurrent ARF on their clinical record summary page (p = 0.004) and had RHD risk category (p < 0.001) and smoking status (p = 0.005) recorded in their clinical record by the second or third years of the study. There was no significant change in the proportion of people with known RHD, and on the register, who had the diagnosis of RHD documented in their clinical record (Table 4).

The proportion of people who had clear documentation of risk classification, and for whom the auditor did not need to apply the algorithm, increased from 56% (87/154) in 2008 to 71% (103/145) in 2009 and 76% (118/156) in 2010 (p < 0.001). This improvement was observed across all health centres, except one where documentation of risk was already 100% in the first year. Documentation of attendance remained essentially unchanged over the three years (Table 4). A record of recent attendance increased in people in high and medium-risk categories, but decreased in people at low or undetermined-risk. These changes were not statistically significant.

### Impact of the CQI process on delivery of care

In analysis of aggregated data across the 6 health centres, we observed improvements for almost all indicators of service delivery (Table 5). The trends were statistically significant for the proportion of people with a record of having received 40% or more of scheduled BPG injections (p = 0.04), who had their BPG injections scheduled at four-weekly intervals (p < 0.001), who had been reviewed by a doctor within the past two years (p = 0.003), for people in high and medium-risk categories who had been reviewed by a doctor within the past six months (p = 0.004), and for people who had received influenza immunisation within the past 12 months (p = 0.001).

**Table 5:** Documented delivery of clinical care

	**Risk classification**^**1**^	**2008**	**2009**	**2010**	**p value**
Received 80% + of scheduled injections	All	25% (29/116)	26% (25/97)	23% (24/103)	0.78
	High/Med	30% (17/57)	32% (14/44)	28% (13/47)	0.83
Received 60% + of scheduled injections	All	42% (49/116)	53% (51/97)	52% (54/103)	0.13
Received 40% + of scheduled injections	All	70% (81/116)	76% (74/97)	82% (84/103)	**0.04**
Frequency of BPG injections scheduled at four weekly	All people with documented requirement for regular BPG injections	20% (23/116)	32% (31/97)	52% (54/103)	<**0.001**
Actions to improve uptake for people who received <80% of injections	Active recall	81% 70/86	94% 68/72	89% 70/79	0.15
	Arrange BPG if out of community	59% 51/86	62% 45/72	63% 50/79	0.60
	Prevention advice	64% 55/86	76% 38/72	39% 31/79	**0.002**
	Family meeting	31% 27/86	17% 12/72	8% 6/79	**<0.001**
	Action plan	28% 24/86	18% 13/72	5% 4/79	**<0.001**
	Additional measures after first active recall (home visits, delivery of written reminders, phone text messages)	27% 23/86	24% 17/72	46% 36/79	**0.01**
Active recall plus at least one other of the above strategies	for people who received <80% of injections	69% 59/86	85% 61/72	72% 57/79	0.56
Echocardiogram	all within three years	55% (85/154)	60% (87/145)	62% (97/156)	0.21
	high and medium risk within 12 months^1^	39% (23/69)	42% (22/52)	44% (27/61)	0.20
Documented review by doctor	All within 2 years	73% (112/154)	83% (121/145)	86% (134/156)	**0.003**
within 6 months	High/medium	46% (34/69)	67% (35/52)	74% (45/61)	**0.004**
within 12 months	Low/undetermined	58% (49/85)	71% (66/93)	66% (63/95)	0.24
Documented dental review	all within 2 years	11% (17/154)	20% (29/154)	16% (25/156)	0.26
	High/medium within 12 months	10% (7/69)	21% (11/52)	18% (11/61)	0.21
Documented review by cardiologist/physician	All within 2 years	51% (78/154)	49% (71/145)	56% (87/156)	0.36
	High/medium within 12 months^2^	39% (27/69)	48% (25/52)	43% (26/61)	0.66
Influenza immunisation within 12 months	All	37% (57/154)	54% (78/145)	55% (86/156)	**0.001**
	High/medium	38% (26/69)	58% (30/52)	61% (37/61)	**0.008**
Pneumovax - at least three doses since birth	All	0% (0/154)	0% (0/145)	13% (21/156)	n/a
	High/medium	0% (0/69)	0% (0/52)	20% (12/61)	n/a
Record of provision of educational materials about rheumatic fever (DVD/video/written materials)	All	6% (9/154)	6% (9/145)	1% (2/156)	0.06
Prescribed warfarin	High/medium	20% (14/69)	31% (16/52)	26% (16/61)	0.41
INR testing	For those on Warfarin (at least two INRs in past 6 months)	100%	100%	100%	n/a
INR result	Of those with test results, % within recommended range	64% (9/14)	69% (11/16)	75% (12/16)	0.52

^1^recommendation is 3–6 monthly echocardiogram for high risk.

^2^recommendation is 6 monthly specialist review for high risk [13].

Bold text is used to highlight p-values of <0.05.

Delivery of secondary prophylaxis was considered to be the most important indicator. The proportion of people receiving ≥80% of scheduled BPG injections did not significantly improve over the duration of the study. Among people receiving <80% of scheduled injections, the proportion who had a record of active recall plus at least one other action taken to improve uptake, remained steady between 69% and 85% during the project. The actions taken to improve BPG uptake changed over time: 'prevention advice’, 'family meetings’ and 'development of action plans’ all significantly decreased. Other actions in addition to the initial recall, such as home visits, further written reminders, phone text messages, significantly increased.

### Assessment of the state of primary health care systems to support best practice in RHD care

Overall SAT scores increased by one point between 2008 and 2010. Tests of statistical significance were not calculated, given the somewhat subjective nature of these scores, and because the purpose of these scores was to engage and provide feedback to clinic staff in the participatory CQI process. Overall scores for individual health centres were all within 2 points of the mean score for all health centres, with a range between 4 and 8 in 2008, 5–8 in 2009 and 5–9 in 2010. Regarding individual domains of the SAT, 'organisational influence and integration’ showed the strongest indication of improvement, from mean 6 points in 2008 to 8 points in 2010. Three system component scores - 'Delivery system design’, 'Self-management support’, and 'Links with the community, other health services and other services and resources’ - showed a one-point increase during the study period. Thus the perceived state of most system components appeared to improve over the three years of the study. The changes in averaged scores from all health centres was not marked for any particular system component, but scores assigned by individual clinics to specific items were seen to shift substantially between baseline and follow-up.

Within the domain 'Links with the community, other health services and other services and resources’, the system item on 'cooperation on regional health planning and resource development’ showed a low baseline score (4 in 2008), and the most marked improvement (8 in 2010) of the SAT scores. The system item with the highest average score across the six health centres was 'client access and cultural competence’; 8 in 2008, 9 in 2010, both scores 2 points above average for all items for each of these years. The systems item which consistently had relatively low scores across the three years with no noticeable improvement was 'infrastructure, supplies and equipment’. The average scores for this item were influenced by one health centre that had particularly low scores (0–2) across all three years of the project.

The factors that were believed to be associated with adequacy of delivery of ARF/RHD-related activities were subjectively assessed by the project officer and other participating investigators; these are listed in Table 6.

**Table 6:** Summary of factors influencing performance of 6 remote NT health centres in delivering services to people with ARF/RHD (Ordered in terms of amenability to change)

**Determinants of relatively good performance**	**Determinants of relatively poor performance**
1. Clear allocation of responsibility for RHD program among health centre staff	1. Patient flows in health centre do not direct RHD clients to staff responsible for RHD care
2. Good regional management – commitment to CQI, resourcing for CQI	2. Lack of clear allocation of responsibility for RHD care
3. Effective feedback and management action in response to feedback from CQI process	3. Lack of effective outreach services
4. Good Aboriginal Health Practitioner involvement in health centre operations	4. Changes and inefficiencies in patient information systems
5. Good outreach arrangements – including drivers, Aboriginal Health Practitioners	5. Lack of regular/stable staffing, including medical practitioner service
6. Public health-oriented chronic disease support from regional level to health centres	6. Health Centre Management turnover, unstable management structure
7. Staff stability and continuity, including availability of experienced GP	7. Larger number of clients, complexities of urban environment

## Discussion

In a challenging clinical environment characterised by sustained high rates of ARF/RHD, we have shown a significant improvement in the delivery of care for people with ARF/RHD in association with implementation of a CQI process based on participatory action research principles. Major findings include improvement in important indicators of clinical care, including delivery of scheduled BPG injections, scheduling of injections at the recommended interval of 4 weeks, and documentation of regular review by a medical specialist. While the gap between actual care delivered and best practice leaves much room for improvement, the successes achieved are a positive development towards attaining better outcomes. Significant improvements in clinical record-keeping relating to ARF/RHD were also achieved.

Yet the proportion of people receiving ≥80% of scheduled BPG did not improve, remaining around 25% across all six health centres over the three years of the study. This is consistent with data on the regional RHD register for the period covered by this study (unpublished data). Thus, although many people were receiving greater percentages of their BPG doses by the end of the study, it is clear that achievement of best practice is a long way off. The impact of the improvement we found (an increase in people getting ≥40% of their scheduled BPG doses) on population RHD burden is difficult to predict, as the relationship between needles received and RHD is non-linear, depending on Group A Streptococcal exposure pressures and strain type in circulation, and host factors. In recent years, there is evidence that overall in the NT, adherence has improved in the lower ranges - fewer people are receiving <50% of injections, more are receiving 50-80%, but without substantial changes to those receiving >80%. While this is not ideal, and is a rationale for further planned interventions to achieve better outcomes, it appears to have correlated with reductions in recurrence rates [46].

A key output of the project has been the development of a clinical audit tool consistent with the 2006 and current National ARF/RHD Guidelines [13,35]. This tool has proved suitable for measuring change in key performance indicators within a CQI process in diverse regional and remote Indigenous primary health care services. It is available via the One21seventy website [32]. The project provided substantial capacity-building opportunities for both Aboriginal and non-Aboriginal staff of the health services engaged in the project. The action research method implemented is an important means of optimising key stakeholder engagement and support for the project from management level to the health centre frontline staff. The ABCD process used in this study has successfully improved the care of people with diabetes mellitus and other chronic diseases in Australian Indigenous primary health care settings [30].

An important but unexpected outcome of the study was the observed reduction in numbers of people requiring ongoing secondary prophylaxis as a consequence of appropriate cessation after specialist review. Adults >21 years of age with low-risk disease who have not had an episode of ARF for the last 10 years do not require ongoing BPG (Table 1). Inappropriate inclusion of people on clinic lists for ongoing BPG generates excessive workloads, potentially detracting from the quality of secondary prophylaxis delivery systems, and exposing people to unnecessary treatment. ARF/RHD under-diagnosis and under-treatment have been flagged as being the major concerns in this environment [13,38], but we have demonstrated that over-treatment is also an important issue and can be reduced by appropriate specialist intervention.

The percentage of charts containing a current penicillin prescription initially rose, then fell again to below baseline in the third year of the study. A transition from paper to electronic prescribing during the study period may have contributed to these changes. Best practice requires an up-to-date script be available, and prescription-writing provides an opportunity for medical review of the file and of the secondary prophylaxis stop date; however, absence of a script does not prevent administration of injections since nurse and Aboriginal health practitioners are authorised to administer BPG injections regardless [39]. The proportion of clients documented to have been provided with educational resources was disappointingly low throughout the study. Improving the provision of culturally-relevant educational information, and the impact this may have on needle uptake, is now the focus of ongoing research. When interpreting the audit data in this report important considerations include: 1) changes in the numbers of people on clinic ARF/RHD lists; 2) changes in the proportion of people identified as requiring regular BPG injections; 3) changes in risk classifications over time. However the changes in these numbers were <10% of the total number of records audited and are unlikely to have significantly influenced findings.

The general challenges of providing good health care in remote Australian Indigenous communities are not unique, but have similarities with those experienced in First Nations and Native American communities in North America/Canada, and Maori communities in New Zealand. Some of the solutions are also similar: e.g. the importance of community control of health service delivery [40,41], whereas other solutions are not: e.g. mobile telephone-based solutions [42,43]. Although phone-based reminders are used where possible, including in this study, this can be hampered by high turnover of telephone numbers in our setting. The specific challenges of ARF/RHD control in Indigenous societies are also not unique to our setting. ARF/RHD burden is disproportionally borne by Indigenous populations globally [44]. The early successes of New Zealand’s ARF/RHD register [28] helped to provide impetus for the creation of the Australian Northern Territory register; RHD registers as the basis for RHD control programs also exist in India, Cuba, Egypt [45] and elsewhere. We are unaware however of literature from non-Australian settings dealing with CQI processes at health service level to improve ARF/RHD control.

Limitations of the study include the lack of a comparison group and lack of multiple observations prior to implementation of the CQI process. However, the study was not intended to demonstrate a causal effect, but rather to describe trends in guideline scheduled services in association with the implementation of a CQI process in the context of very limited knowledge on adherence to best practice guidelines for RHD care. The clinical audits rely on documentation of information in health centre records on the understanding that, if information is not clearly documented, it is also unavailable to health staff responsible for delivering care. Clinical audit data are not necessarily an accurate reflection of the actual care delivered; nevertheless they provide accessible, measurable indicators likely to be closely associated with quality of care. Assessment of adherence to guidelines relies upon accurate RHD risk classification. The appropriate risk category in each case was not always clear from the clinical record, but all efforts were made to assign correct risk level, based on clinical and echocardiographic information, when documentation was unavailable. Calculation of 'days at risk’ (the interval between 28 days from the last penicillin dose and receipt of the next injection) was not assessed as it was not included in RHD audits at the time this research was conducted (2008–2010). Finally, younger people in the initial years after an episode of ARF are most important to target for improved adherence strategies; local data shows highest recurrence rates in the first year after ARF diagnosis [46]. We did not analyse the effect of the CQI intervention according to age or time elapsed since ARF diagnosis; however, the study aimed to assess overall impacts on clinical performance, and our ongoing research aims to determine differential effects of interventions by age and duration since ARF diagnosis.

The operation of health centres was reflected somewhat in their SAT scores. The component assessing 'organisational influence and integration’ improved over 3 years, and appeared to be related to performance in BPG prophylaxis. Although the SAT scores were inherently subjective, the project officer monitored and assisted in the assignment of test scores for consistency and results were chiefly used to engage clinic staff through the process of self-assessment.

While the data presented in this report cannot be regarded as being representative of all health centres in the region, they help us to understand the wide differences in quality of RHD care between health centres and possible contributing factors. There is a clear need to identify and address obstacles in the way of best practice. Of course, solutions must also be tailored to the specific needs of each clinic. A substantial cause for optimism is the high degree of engagement shown by people with ARF/RHD, with up to 90% attending the clinic within a three-month period, usually for ARF/RHD care. Such high rates of attendance provide important opportunities for staff to improve the quality of care, particularly adherence with secondary prophylaxis.

## Conclusion

In the absence of effective group A streptococcal vaccines, primordial prevention offers a key long-term answer for ARF/RHD in Aboriginal Australians and Torres Strait Islanders, especially those living in remote communities. In the meantime, secondary prevention is the best cost-effective option, but this has proven to be difficult to deliver. Key factors include evidence-based guidelines, well-resourced ARF/RHD register programs and sustainable systems for effective service delivery at the primary care level. The systems-directed quality improvement approach described here is an important step towards enhancing the prevention and management of ARF/RHD in people at highest risk. The CQI process provides a mechanism for engagement of individual practitioners, health centre teams and those responsible for management and delivery of care at regional and national levels. It identifies priority areas that need attention. To be most effective, any CQI process must be supported at a system-wide level, with good leadership and management. Our findings point to the need for a stronger focus on improving the uptake of scheduled BPG injections. Any missed injection is a major concern. As such, further CQI research should now focus on the most effective interventions, taking in account diverse community conditions, and sustainability.

## Competing interests

The authors declare that they have no competing interests.

## Authors’ contributions

APR drafted the final manuscript; RSB provided overall leadership and oversight of the project, prepared the first draft and oversaw the analyses. MF was the project officer who chiefly implemented the study. RSB, MIM, KNE, JRC conceived, designed and helped to implement the study. RS, BG, SN, MD, TC, MGP and MC comprised the Project Management Committee; they actively participated in conducting the study and contributed to the manuscript. All authors approved the final manuscript.

## Pre-publication history

The pre-publication history for this paper can be accessed here:

http://www.biomedcentral.com/1472-6963/13/525/prepub

## Supplementary Material

Additional file 1 **Content of acute rheumatic fever and rheumatic heart disease clinical audit tool *(formatted here as a list due to space constraints.*** See http://www.one21seventy.org.au*for formatted version created for data collection).* Click here for file
