# Supplementary material for: Improvement in rheumatic fever and rheumatic heart disease management and prevention using a health centre-based continuous quality improvement approach
Source: BMC Health Serv Res. 2013 Dec 18;13:525. doi: 10.1186/1472-6963-13-525 (PMC3878366; doi:10.1186/1472-6963-13-525)
Supplement: Additional file 1 — Content of acute rheumatic fever and rheumatic heart disease clinical audit tool (formatted here as a list due to space constraints. See http://www.one21seventy.org.aufor formatted version created for data collection). [file 1472-6963-13-525-S1.docx]

| **1.1** Person ID  **1.2** Medicare number recorded in notes  **1.3** Date of Birth  **1.4** Age at date of audit  **1.5** Sex  **1.6** Indigenous status: Aboriginal /Torres Strait Islander / Both / Neither / Not stated  **1.7** Auditor’s initial & surname  **1.8** Audit Date  **Section Two:** Attendance at Health Centre  **2.1** Date last attended  **2.2** Location of record of date last attended: Yes / No  Medical record paper / electronic / both  **2.3** Reason for last attendance:   - - - - Acute care Benzathine penicillin injection / ARF-RHD prophylaxis with oral medication / Well person’s check / Specialist review / Other   **2.4** First seen by:   - - - - Aboriginal &/or Torres Strait Islander Health Worker / Nurse / Doctor / Specialist / Allied health professional / Other / Not Stated   **2.5** If client not seen in last 12 months is there any record of unsuccessful follow-up attempt since last attendance?  Yes / No  **3.1** Is there a record of the following diagnoses on the Health Summary Sheet?   - - - - Definite or suspected acute rheumatic fever (first episode) / Recurrent or suspected recurrent acute rheumatic fever / Rheumatic heart disease       - 3.2 If diagnoses not recorded on Health Summary Sheet, are diagnoses recorded elsewhere in Medical Record?       - Definite or suspected acute rheumatic fever (first episode)       - Recurrent or suspected recurrent acute rheumatic fever       - Rheumatic heart disease   **3.3** Where in the client’s medical record/s is the   - - - - client’s RHD category (according to the RHD register) recorded?       - On Health Summary Sheet / Elsewhere in Medical Record /       - Not Recorded   **3.4** If recorded, what is the client’s risk classification? ____   - 1. If not recorded, what is the classification according   to the National Heart Foundation of Australia and  the Cardiac Society of Australia and New Zealand Guidelines?   - - - - High risk / Medium risk / Low risk / unable to determine / NA   **3.6** Is there a current ARF/RHD Management Plan present?   - - - - Yes / No   1. Smoking status recorded      - - Yes / No   **3.8** What is the recorded smoking status   - - - - Smoker / Non-Smoker / Not recorded   **3.9** What is the recorded alcohol use status   - - - - Unable to determine / Higher risk / Low risk / Risk level not stated / No alcohol | **3.10** If the client’s classification is High Risk, is there documentation indicating prior cardiac surgery? Yes / No  **3.11** If the client’s classification is High Risk, is there documentation in the file indicating the client is awaiting cardiac surgery? Yes / No  **3.12** If the client’s classification is High Risk or Medium Risk, is the client currently prescribed Warfarin? Yes / No  **3.13** If the client is prescribed Warfarin, please record the two most recent INRs including results and dates of these tests.  INR 1 / INR 2  **4.1** Is the client prescribed regular benzathine penicillin injections?   - - - - Yes / No   **4.2** Is the client prescribed oral antibiotic prophylaxis for rheumatic fever instead of benzathine penicillin injections?   - - - - Yes / No   **4.3** Is there a current prescription for benzathine penicillin injections?   - - - - Yes / No   **4.4** Where in the Medical Record is the planned frequency of injections recorded?   - - - - Current prescription / Non-current prescription / Elsewhere in medical record / Not recorded / N/A   **4.5** Is the planned frequency of injections recorded in a clinic master chart?   - - - - Yes / No   **4.6** If recorded in both the client’s medical record and the clinic master chart, are the two records consistent?   - - - - Yes / No   **4.7** If not consistent, which one is currently used?   - - - - Medical record / Clinic master chart / N/A  \| - - - - *Please record the number of benzathine penicillin injections given over the last 12 months.*       - ***Note:*** *a client on* ***MONTHLY*** *injections should have* ***12*** *injections in 12 months;*       - *a client on* ***4 WEEKLY*** *injections should have* ***13*** *injections in 12 months;*       - *a client on* ***3 WEEKLY*** *injections should have* ***17*** *injections in 12 months.* \| \| --- \|   **4.8** Frequency of injections **planned**   - - - - Monthly / 4-weekly / 3-weekly / other / not recorded / NA   **4.9** Number of injections **given** in last 12 months: _____  **4.10** If injections commenced within the last 12 months, record date of first injection  **4.11** Calculate the percent of planned injections that were received in the last 12 months  (or since beginning BPG injections)? **% *(see protocol for calculation method)***  **4.12** If the client has received **fewer than 80%** of planned benzathine penicillin injections, is there a record of:   - an attempt at active recall? - an attempt to contact the relevant health centre to arrange for benzathine penicillin to be given if the client is known to be out of the community? - advice about importance of preventing recurrent ARF? - a family meeting? - an action plan made? - other appropriate action? - Details of other appropriate action:__________ | **4.13** Number of **recorded episodes** of recurrent rheumatic fever in the last 12 months:  **4.14** If ≥1 episode of recurrent rheumatic fever recorded in the last 12 months despite good delivery of benzathine  penicillin (80% or more of scheduled), Is there a record of:   - change to more frequent benzathine penicillin injections? - advice on the role of throat and skin infections in leading to ARF? - advice on the role of overcrowding in predisposing to ARF? - action plan made? - referral to support services (for example, environmental health services, housing services)? other appropriate action? - Details: _________________________  \| - - - - ***Doctor Review*** *is recommended 6 monthly for High Risk and Medium Risk clients and yearly for Low Risk clients.*       - ***Cardiologist/physician/ paediatrician review*** *is recommended 6 monthly for High Risk clients, yearly for Medium Risk clients.*       - ***Echocardiogram*** *is recommended 6 monthly for High Risk clients, yearly for Medium Risk clients, 2 yearly for Low Risk clients*       - *under 15 years and 3 yearly for Low Risk clients over 15 years.*       - ***Influenza vaccination*** *is recommended yearly for High Risk and Medium Risk clients.*       - ***Dental review*** *is recommended yearly for High Risk and Medium Risk clients.*       - ***Polysaccharide pneumococcal vaccination (Pneumovax 23)*** *is recommended for High Risk and Medium Risk clients.* \| \| --- \|   **5.1** Is there a **record of each of the following services** having been provided within the timeframes shown:   - - - - Doctor review (within 2 years) /Cardiologist/ physician/ paediatrician review (within 2 years) / Echocardiogram (within 3 years) /Influenza vaccination (within 2 years) / Dental review (within 2 years) / Polysaccharide pneumococcal vaccination (Pneumovax 23)       - (record 3 most recent immunisations)   **5.2 Education.** Is there a record of the following education about rheumatic fever having been provided?   - - - - Watched DVD or video / Given written materials   1. **Brief intervention.** Is there a record of brief intervention including the following risk factors having been provided?      - - Smoking / Nutrition / Alcohol / Physical Activity |
| --- | --- | --- | --- | --- |
